# Periconceptional Folic Acid Supplementation and Newborn Birth Weights

**DOI:** 10.3389/fped.2022.844404

**Published:** 2022-04-28

**Authors:** Jing Lin, Cheng Wang, Sisi Li, Jie Zhang, Lei Jin, Mingkun Tong, Wenying Meng, Aiguo Ren, Lei Chen, Lei Jin

**Affiliations:** ^1^Department of Obstetrics and Gynecology, The Sixth Medical Center of People's Liberation Army (PLA) General Hospital, Beijing, China; ^2^The Second School of Clinical Medicine, Southern Medical University, Guangzhou, China; ^3^Institute of Reproductive and Child Health, Peking University/Key Laboratory of Reproductive Health, The National Health Commission of the People's Republic of China, Beijing, China; ^4^Department of Epidemiology and Biostatistics, School of Public Health, Peking University, Beijing, China; ^5^Tongzhou Maternal and Child Health Care Hospital of Beijing, Beijing, China

**Keywords:** folic acid, micronutrients, vitamin, birth weight, pregnancy, small-for-gestational-age, large-for-gestational-age

## Abstract

**Background:**

The relationship between maternal folic acid supplementation and the birth weights of offspring remains inconclusive.

**Aim:**

To examine the associations between maternal supplementation with folic acid only (FAO) or multiple micronutrients containing folic acid (MMFA) and newborn birth weights, as well as the risk of small for gestational week age (SGA) and large for gestational week age (LGA) newborns.

**Methods:**

Data on 31,107 births from 2015 to 2018 were extracted from the population-based prenatal health care system in a district of Beijing. Generalized linear and logistic regression models were used to evaluate the association between maternal periconceptional folic acid supplementation and birth weights or with risk of small for gestational week age (SGA) and large for gestational week age (LGA).

**Results:**

Compared with newborns whose mothers did not use any folic acid supplements, the newborns with maternal periconceptional folic acid supplementation had similar median birth weight but had a lower risk of SGA [adjusted odds ratio (*aOR*) = 0.81 (*95% CI*: 0.68–0.97)], however newborns born to mothers who took multiple micronutrients with folic acid (MMFA) with high compliance had a 25.59 g (95% *CI:* 6.49–44.69) higher median birth weight. Periconceptional women took folic acid only (FAO) (a*OR* = 0.83; 95%*CI:* 0.67–1.01) or MMFA (a*OR* = 0.74; 95%*CI*: 0.60–0.91) with high compliance decreased the risk of SGA, but has no impact on the risk of LGA.

**Conclusion:**

Periconceptional FAO supplementation has no impact on the median birth weight of offspring and the risk of LGA. Compared with FAO, MMFA supplementation may increase the average birth weight, and a high compliance of supplementation with FAO or MMFA may reduce the risk of SGA, with MMFA having ad stronger effect than FAO.

## Impact

Periconceptional FAO supplementation has no impact on the median birth weight of offspring and the risk of LGA.; Compared with FAO, MMFA supplementation increases the average birth weight, and a high compliance of supplementation with FAO or MMFA reduces the risk of SGA, with MMFA having ad stronger effect than FAO.

- Periconceptional folic acid only supplementation may have no impact on the average birth weight and the risk of LGA of offspring.
- Periconceptional multiple micronutrients supplementation may increases the average birth weight compared with folic acid only.
- A high compliance of supplementation with multiple micronutrients may reduce the risk of SGA.

## Introduction

Birth weight has long been used as an indicator for the healthy status of newborns and it is widely accepted that an abnormal birth weight, such as SGA or LGA, can have either short or long-term effects on an offspring's health (1–7). Presently, while the prevalence of SGA and LGA in China is at 5.74% (8) and 16.21% (9), respectively, the prevalence of SGA and LGA has also decreased significantly in recent years (10).

Folate is a water-soluble member of the vitamin B complex family. It acts as a substrate and cofactor in many cellular metabolic pathways and biochemical reactions, such as DNA and RNA synthesis, intracellular signal transduction and methylation (11–13). In the 1990s, large-scale studies confirmed that in maternal periconceptional supplementation with folic acid FA, (the synthetic form of folate), only FAO can prevent neural tube defects (14, 15). The Food Fortification Initiative 2017 report lists 86 countries that mandate iron or folic acid fortification of one or more cereal grains (16). Based on the situation in China, China's Ministry of Health launched a nationwide program in 2009 to increase the folic acid intake of women to prevent neural tube defects (NTDs), and the program provides folic acid supplements (0.4-mg folic acid per tablet) free of charge to women who intend to get pregnant (17). Since this program began, the prevalence of FAO supplements had gradually increased in China, with some women supplementing with multiple micronutrients containing folic acid (MMFA) that they pay for themselves. Currently, the prevalence of maternal periconceptional folic acid supplementation is 90.08% in Beijing (18).

Evidence that FAO or MMFA can prevent neural tube defects has also stimulated research on the relationship between maternal folic acid supplementation and other pregnancy outcomes. Among these studies, the subject of most concern is the effect of folic acid supplementation on birth weights. However, the evidence on the effect of folic acid supplementation on the birth weights of newborns is not consistent in the literature (19–21). Retrieving the literature from China and other countries, the generation R study in Rotterdam found that periconceptional folic acid supplementation could promote fetal growth, lead to birth weight gains, and decrease the risk of SGA (19). A meta-analysis combining the data from the re-analysis done in the Aberdeen folate supplementation trial and an update of the Cochrane review concluded that maternal folic acid supplementation did not affect the average birth weight, but that high doses of folic acid may lower the risk of having a low birth weight (LBW) in newborns (20). However, a multi-center cohort study based on the Spanish population suggested that the periconceptional use of medium (1 mg/day) and high-dose (>1 mg/day) folic acid supplements is associated with decreased birth weights (21). Most of these studies focused on whether the women took folic acid supplements and the effect of the folic acid supplement dosage on birth weights, but rarely considered the formula of folic acid supplements (FAO or MMFA) the way of taking the folic acid supplements, including the time of initiation (before or after conception) and the compliance with taking folic acid supplements. In our previous study, based on the monitoring data of routine pregnancy care and data on birth defects of women delivering their newborns in the Tongzhou District of Beijing from 2013 to 2018, we found that the high compliance use rate for periconceptional supplementation was only 41.5% (18).

Folate metabolism is particularly active during cell division, and embryo tissues are very sensitive to folate deficiency or metabolic changes (22, 23). The folate status of the human body is affected by heredity, the environment, and eating habits. Thus, in many developed countries, folate deficiency may be relatively rare, but a sub-optimal folate status is commonly encountered (24). The perinatal period is also an important period of cell division and embryo development. Although folic acid supplementation during pregnancy is the standard care for expectant pregnant women, not all expectant pregnant women start taking folic acid supplementation for more than 80% of the total number of days in the first 3 months of pregnancy. Therefore, the time when pregnant women initiate taking folic acid supplements and the frequency of their taking these folic acid supplements may be the key factors for folic acid affecting the birth weights of their offspring.

The study aimed to understand the use of folic acid supplements in Beijing women during the periconceptional period, and to evaluate its impact on birth weights and the risk for SGA and LGA for their offspring, to further understand the safety and necessity of maternal periconceptional folic acid supplementation.

## Materials and Methods

### Data Sources

The data was drawn from the Prenatal Health Care System (PHCS) at the Tongzhou Maternal and Child Health Care Hospital in Beijing from 2015 to 2018. The PHCS is a networked system in Beijing City, and it covers all the community hospitals and hospitals with obstetrics and gynecology. Each woman who is diagnosed as pregnant in a hospital needs to register with the PHCS before 13 weeks of pregnancy at the community hospital closest to their place of residence. A prenatal health care booklet with a barcode containing a unique 14-digit number is given to the women, which is her pregnancy identification number in the system. She can be identified by scanning the barcode when she goes to a delivery hospital in Beijing to have her prenatal health care examinations, and then her clinical medical information will be recorded in the system.

Uniformly trained doctors or nurses from the community hospital collected the demographic information, medical history, obstetrics and gynecology history, disease history, and information on folic acid supplementation during the periconceptional period using face-to-face interviews at the time of registration. The information includes maternal nationality, age, education level, occupation, pre-pregnancy body mass index (BMI), compliance with supplementation, delivery year, household registration, and information on folic acid supplementation.

The information on the gestational week of delivery, birth weight, gender, and other delivery information for the baby was entered in the PHCS by the doctors and nurses at the delivery hospital. The birth weight was measured by the obstetrician or midwife of the delivery hospital using an electronic weighing scale with an accuracy of 10 g <30 min after the baby was delivered.

From October to November every year, 200 women were selected monthly by the doctors at the District Maternal and Child Health Hospital, and the data in the pregnancy health database was verified by a telephone survey. The qualifying rate for the information from the annual information verification was more than 96.5%. The information on the newborns included birthweight, gender, and other parameters, as well as information on gestational complications, including gestational diabetes, hypertensive disorders of pregnancy, and anemia, among others. This information was then checked with the clinical medical records information obtained from the Maternal and Child Health Hospital's clinic information system in this study.

### Participant Selection

**Figure 1:**
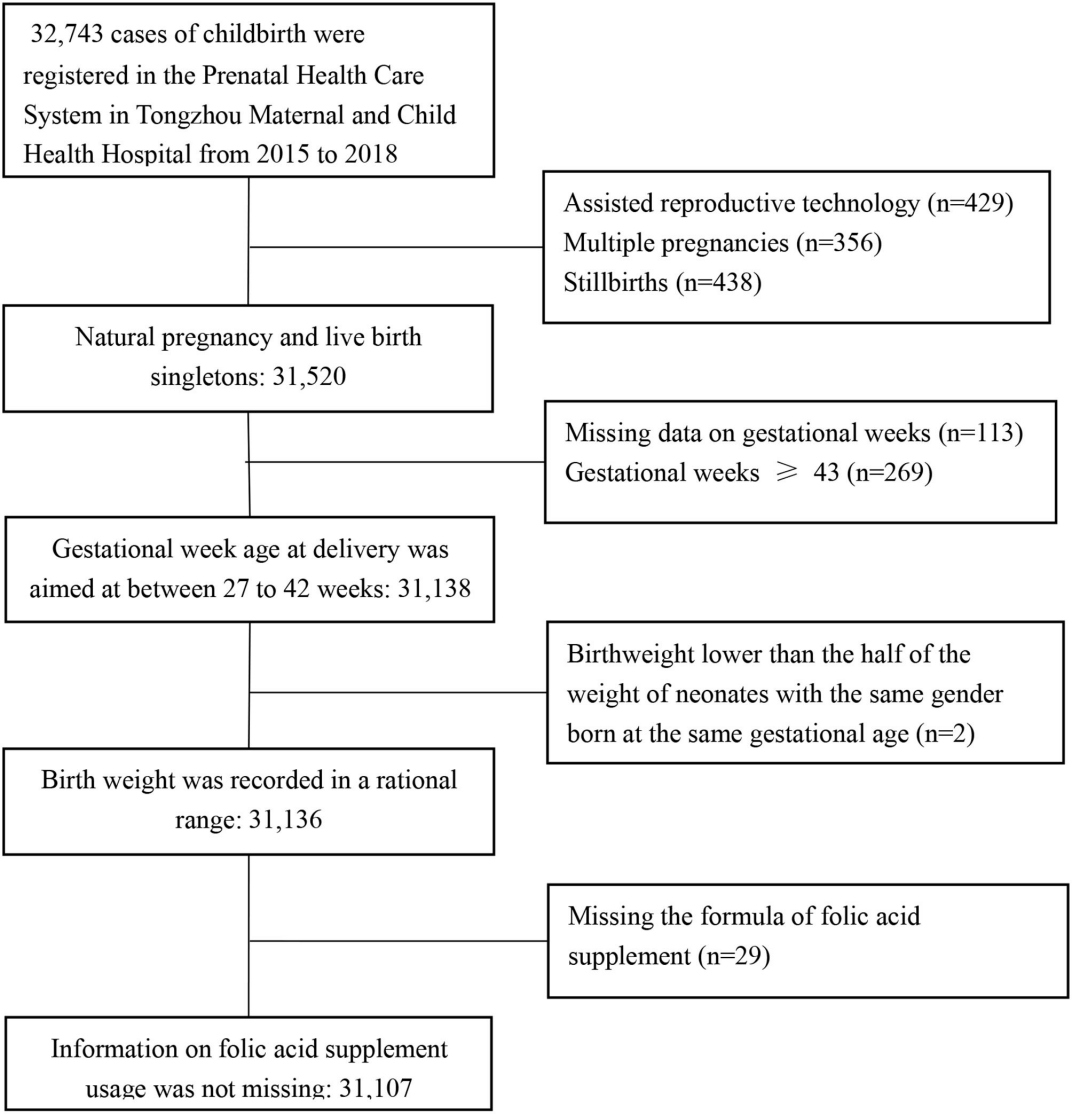
Chart for the selection of participants.

In total, 32,743 cases of childbirth were registered in the PHCS of the Tongzhou Maternal and Child Health Hospital from January 1, 2015 to December 31, 2018. We excluded pregnancies achieved by assisted reproductive technology (*n* = 429), multiple pregnancies (*n* = 356), stillbirths (*n* = 438), those with missing data on gestational weeks (*n* = 113), with gestational weeks >43 (*n* = 269), and with a birthweight lower than half of the weight of neonates with the same gender and gestational age (*n* = 2), or missing information on formula of folic acid supplementation (*n* = 29). As a result, 31,136 records for mother and newborn pairs were left for analysis in the study (Figure 1).

This study was approved by the Institutional Review Board of Peking University (No.: IRB00001052-18010).

### Definition of Periconceptional FA Supplementation

Information maternal on the use of periconceptional folic acid supplements was collected with four questions: Did you take folic acid supplements during periconceptional period? (Yes/No); Which kind of formula supplements did you take? [Pills containing folic acid only (FAO) or multiple micronutrients containing folic acid (MMFA)]; What time did you initiate the supplementation? (before the last menstrual period or after the last menstrual period); What was the frequency of your supplementation? (8 days or more out of 10 days or <8 days out of 10 days). The last menstrual period was used to determine the timing of conception, pre-conception was indicated by “before the last menstrual period” and post-conception was indicated by “after the last menstrual period.” The periconceptional period was defined as the period from 12 weeks before the last menstrual period to 12 weeks after the last menstrual period. Taking folic acid supplements with high compliance was defined as supplementation for 8 days or more out of 10 days, whereas low compliance was defined as supplementation for <8 days out of 10 days.

### Definition of SGA and LGA

The outcome variables in this study are birth weight, SGA, and LGA, which are defined as follows: SGA: according to the Chinese male and female birth weight percentile in 2015 (25), SGA is defined as a newborn whose birth weight is lower than the 10th percentile of the same sex and gestational week age in the reference population, while LGA is defined as a newborn whose birth weight is higher than the 90th percentile of the same sex and gestational week age in the reference population.

### Statistical Analyses

Since birth weight did not conform to the normal distribution (*P* < 0.001), the median and interquartile range [lower quartile (*P*_25_), upper quartile (*P*_75_)] were selected to describe the average and deviation in birth weight. The descriptive statistics for the categorical variables were reported as frequencies and percentages. The chi-square test was used to compare the general characteristics of women among the groups of women with using folic acid supplements. The Wilcoxon rank-sum test was used to compare the differences in average birth weight of different genders. Spearman's rank correlation was used to analyze birth weight change by years.

Wilcoxon rank-sum test, Kruskal–Wallis *H* test, and generalized linear model were used to analyze the relationship between newborn birth weight and maternal folic acid supplementation during periconception. The chi-square test, chi-square test for trends, and logistic regression model were used to analyze the relationship between the prevalence of SGA and LGA and maternal folic acid supplementation during periconceptional period. Two-sided values of *P* < 0.05 were considered statistically significant. All data was analyzed using SPSS 24.0 (IBM, Armonk, NY, USA).

## Results

### Maternal and Newborn General Characteristics

The maternal mean (SD) age was 29.16 (±4.06) years, the mean (SD) pre-pregnancy BMI was 22.21 (±3.43) kg/m (2), and the mean (SD) gestational week age was 39.19 (±1.46) weeks. The general characteristics of the mothers and newborns among the compared groups is shown in Table 1. The differences in the proportion distribution of age, education level, occupation, household registration, pre-pregnancy BMI, parity, delivery years, gestational diabetes, and anemia in the FAO, MMFA, and Non-FA (NFA) groups were statistically significant (*P* < 0.05).

**Table 1:** General characteristics of mothers and newborns by folic acid supplement type in singleton births at the Tongzhou Maternal and Child Health Hospital, 2015–2018^a^.

**Characteristics**	**Total *N* = 31,107 *n* (%)**	**NFA *N* = 2,810 *n* (%)**	**FAO *N* = 11,600 *n* (%)**	**MMFA *N* = 16,697 *n* (%)**	***P*-value**
Ethnicity					**0.070**
Han	29,250 (94.0)	2,661 (94.7)	10,933 (94.2)	15,656 (93.8)	
Other	1,857 (6.0)	149 (5.3)	667 (5.8)	1,041 (6.2)	
Age/years					**<0.001**
16	3,381 (10.9)	432 (15.4)	1,222 (10.5)	1,727 (10.3)	
25	14,437 (46.4)	1,158 (41.2)	5,504 (47.4)	7,775 (46.6)	
30	9,919 (31.9)	882 (31.4)	3,638 (31.4)	5,399 (32.3)	
35–48	3,370 (10.8)	338 (12.0)	1,236 (10.7)	1,796 (10.8)	
Education					**<0.001**
Junior high school and below	3,184 (10.2)	559 (20.3)	1,291 (11.2)	1,334 (8.0)	
High school or equivalent	6,092 (19.6)	755 (27.5)	2,332 (20.2)	3,005 (18.1)	
College school	9,671 (31.1)	756 (27.5)	3,574 (31.0)	5,341 (32.2)	
Bachelor degree or above	11,934 (38.3)	677 (24.6)	4,325 (37.5)	6,932 (41.7)	
Occupation					**<0.001**
Manager of a unit	2,825 (9.1)	305 (11.5)	936 (8.2)	1,584 (9.6)	
Professional	6,339 (20.4)	329 (12.4)	2,482 (21.8)	3,528 (21.5)	
Clerical personnel	4,721 (15.2)	305 (11.5)	1,615 (14.2)	2,801 (17.0)	
Commercial or service personnel	5,962 (19.2)	442 (16.7)	2,244 (19.7)	3,276 (19.9)	
Unemployed	4,820 (15.5)	541 (20.4)	1,872 (16.5)	2,407 (14.6)	
Other occupations	5,783 (18.6)	728 (27.5)	2,219 (19.5)	2,836 (17.3)	
Household registration					**<0.001**
Couples both with non-local	12,633 (40.6)	1,110 (39.5)	4,697 (40.5)	6,826 (40.9)	
Only the wife with local	12,880 (41.4)	1,162 (41.3)	4,640 (40.0)	7,078 (42.4)	
Only the husband with local	5,594 (18.0)	538 (19.1)	2,263 (19.5)	2,793 (16.7)	
Pre-pregnancy BMI (kg /m^2^)					**0.003**
Lean (<18.5)	3,484 (11.2)	303 (10.8)	1,293 (11.2)	1,886 (11.3)	
Normal-weight (18.5–24.0)	19,571 (62.9)	1,695 (60.4)	7,346 (63.4)	10,530 (63.2)	
Over-weight (24.0–28.0)	6,052(18.3)	585 (20.7)	2,240 (19.3)	3,227 (19.2)	
Obesity (28.0 or more)	1,955 (7.4)	222 (8.0)	704 (6.1)	1,029(6.3)	
Gestational week age /weeks					**0.207**
27	1,152 (3.7)	118 (4.2)	439 (3.8)	595 (3.6)	
37	6,754 (21.7)	652 (23.2)	2,478 (21.4)	3,624 (21.7)	
39	18,339 (59.0)	1,605 (57.1)	6,877 (59.3)	9,857 (59.0)	
41–43	4,862 (15.6)	435 (15.5)	1,806 (15.6)	2,621 (15.7)	
Parity					**<0.001**
Primiparity	16,677 (53.6)	1,152 (41.3)	6,378 (56.8)	9,147 (56.2)	
Multiparity	13,625 (43.8)	1,635 (58.6)	4,849 (43.2)	7,141 (43.8)	
Delivery year					**<0.001**
2015	5,273 (16.9)	689 (24.5)	2,020 (17.4)	2,564 (15.4)	
2016	8,902 (28.6)	630 (22.4)	3,760 (32.4)	4,512 (27.0)	
2017	8,746 (28.1)	768 (27.3)	3,181 (27.4)	4,797 (28.7)	
2018	8,186 (26.3)	723 (25.7)	2639 (22.8)	4,824 (28.9)	
Gestational diabetes					**<0.001**
Yes	7,979 (25.6)	633 (22.5)	2,977 (25.7)	4,369 (26.2)	
No	23,128 (74.4)	2,177 (77.5)	8,623 (74.3)	12,328 (73.8)	
Hypertensive disorders of pregnancy					**0.048**
Yes	960 (3.1)	104 (3.7)	372 (3.2)	484 (2.9)	
No	30,147 (96.9)	2,706 (96.3)	11,228 (96.8)	16,213 (97.1)	
Thyroid disease					0.128
Yes	1,441 (4.6)	121 (4.3)	573 (4.9)	747 (4.5)	
No	29,666 (95.4)	2,689 (95.7)	11,027 (95.1)	15,950 (95.5)	
Anemia					**<0.001**
Yes	6,232(20.2)	688 (24.8)	2,423(21.1)	3,121 (18.8)	
No	24,592(79.8)	2,084 (75.2)	9,069 (78.9)	13,439 (81.2)	
Liver disease					0.390
Yes	571 (1.8)	60 (2.1)	216 (1.9)	295 (1.8)	
No	30,536 (98.2)	2,750 (97.9)	11,384 (98.1)	16,402 (98.2)	

a *Missing values n (%) Education level 235 (0.8%), Occupation 667 (2.1%), Pre-pregnancy BMI 58 (0.2%), Parity 805 (2.6%), Anemia 269 (0.9%)*.

*BMI calculated by the formula BMI = weight (kgs)/height (m^2^). Bold values represents p < 0.05*.

### Periconceptional FA Supplementation

The prevalence of folic acid supplementation was 91.0%. Among women who took folic acid supplements during the periconceptional period, 41.0% took FAO and 59.0% took MMFA. The proportion of women who took FAO and initiated supplementation before the last menstrual period was 59.6%, compared with 40.6% for women who took MMFA. The proportion of women who took FAO with high compliance was 53.8%, while this proportion was 36.7% for MMFA (Table 2).

**Table 2:** Folic acid supplementation among women who gave birth to singleton newborns at the Tongzhou Maternal and Child Health Hospital, 2015–2018.

**Folic acid supplementation**	***n***	**(%)**
Folic acid supplementation	**31,107**	
Yes	28,297	91.0
No	2,810	9.0
Formula	**28,297**	
FAO	11,600	41.0
MMFA	16,697	59.0
Initiation time
FAO^a^	**11,599**	
Before conception	6,918	59.6
After conception	4,681	40.4
MMFA	**16,697**	
Before conception	6,781	40.6
After conception	9,916	59.4
Supplement frequency
FAO	**11,600**	
8 days or more out of 10 days	6,244	53.8
<8 days out of 10 days	5,356	46.2
MMFA^b^	**16,694**	
8 days or more out of 10 days	6,129	36.7
<8 days out of 10 days	10,565	63.3

a *The number of pregnant women taking FAO with missing time: 1*.

b *The number of pregnant women with missing frequency of taking MMFA: 3. Bold values represent the total number in the group*.

### Birth Weight, SGA, and LGA

As can be seen in Table 3, the overall median (*P*_25_-*P*_75_) birth weight was 3,390 g (3,110–3,670 g), among which the median (*P*_25_-*P*_75_) birth weight for females was 3,330 g (3,070–3,600 g) and 3,440 g (3,170–3,730 g) for males. According to the Wilcoxon rank-sum test analysis, the differences in the average birth weight of male and female newborns was statistically significant (*P* < 0.001). In addition, according to the Spearman's rank correlation analysis, the median birth weight increased from 2015 to 2018 (*P*_trendtest_ = 0.001).

**Table 3:** Birth weight and prevalence of SGA or LGA among newborns by genders and delivery years.

**Items**	***n***	**Birth weight (g)**					**SGA**		**LGA**	
		**$\bar{X}\pm$S**	***P*_25_**	***P*_50_**	***P*_75_**	***P*-value**	***n* (%)**	***P*-value**	***n* (%)**	***P*-value**
Total	31,107	3,387 ± 454	3,110	3,390	3,670		1,714 (5.5)		3,612 (11.6)	
Gender						**<0.001**		0.385		0.359
Female	15,072	3,332 ± 442	3,070	3,330	3,600		813 (5.4)		1,776 (11.8)	
Male	16,035	3,440 ± 460	3,170	3,440	3,730		901 (5.6)		1,836 (11.4)	
Delivery year						**0.001**		0.376		**0.002**
								0.897^*^		0.014^*^
2015	5,273	3,406 ± 455	3,140	3,400	3,690		274 (5.2)		619 (11.7)	
2016	8,902	3,382 ± 449	3,110	3,380	3,670		520 (5.8)		953 (10.7)	
2017	8,746	3,379 ± 453	3,100	3,380	3,660		478 (5.5)		1,012 (11.6)	
2018	8,186	3,390 ± 461	3,110	3,390	3,680		442 (5.4)		1,028 (12.6)	

* *Chi-square test for trend. Bold values represents p < 0.05*.

The overall prevalence of SGA and LGA were 5.5 and 11.6%, respectively. The differences in prevalence for SGA among newborns by gender or delivery year were not statistically significant. The differences in prevalence for LGA was not statistically significant for gender, but the difference was statistically significant for different delivery years (*P* = 0.002) and the prevalence of LGA increased from 2015 to 2018 (*P*_trendtest_ = 0.014).

### Periconceptional FA Supplementation and Birth Weight

The results of a univariate analysis on the relationship between folic acid supplementation and newborn birth weight are shown in Table 4. According to Wilcoxon rank-sum test and the Kruskal–Wallis *H* test results, the differences in median birth weight between folic acid supplement users and non-users, initiated supplementation pre or post conception, and taking the supplements with high or low compliance, were not statistically significant, but the difference between the median birth weight for the FAO and MMFA groups was statistically significant (*P* = 0.007).

**Table 4:** Periconceptional folic acid supplementation and the birth weight of newborns as well as risk of SGA or LGA, univariate analysis.

**Folic acid supplementation**	***n***	***P*_50_ (*P*_25_-*P*_75_)**	***P*-value**	**SGA**			**LGA**		
				**n (%)**	***χ^2^***	***P*-value**	***n* (%)**	***χ^2^***	***P*-value**
Folic acid supplementation			0.776		2.22	0.137		2.14	0.143
Yes	28,297	3,390 (3,110, 3,670)		1,542 (5.4)			3,264 (11.5)		
No	2,810	3,390 (3,110, 3,670)		172 (6.1)			350 (12.5)		
Formula			0.007		1.01	0.315		3.19	0.074
MMFA	16,697	3,390 (3,120, 3,680)		891 (5.3)			1,972 (11.8)		
FAO	11,600	3,380 (3,110, 3,660)		651 (5.6)			1,290 (11.1)		
Initiation time
FAO			0.122		0.52	0.473		0.54	0.463
Before conception	6,918	3,370 (3,100, 3,650)		397 (5.7)			781 (11.3)		
After conception	4,681	3,390 (3,120, 3,670)		254 (5.4)			508 (10.9)		
MMFA			0.149		0.19	0.667		0.19	0.665
Before conception	6,781	3,380 (3,110, 3,680)		368 (5.4)			792 (11.7)		
After conception	9,916	3,400 (3,130, 3,680)		523 (5.3)			1,180 (11.9)		
Supplement frequency
FAO			0.443		1.37	0.505		4.32	0.116
					0.28^*^	0.597^*^		1.63^*^	0.201^*^
8 days or more out of 10 days	6,244	3,370 (3,100, 3,650)		357 (5.7)			704 (11.3)		
<8 days out of 10 days	5,356	3,380 (3,110, 3,670)		294 (5.5)			586 (10.9)		
None	2,810	3,390 (3,110, 3,670)		172 (6.1)			350 (12.5)		
MMFA			0.656		3.00	0.223		1.04	0.596
					2.21^*^	0.137^*^		0.26^*^	0.610^*^
8 days or more out of 10 days	6,129	3,390 (3,120, 3,680)		172 (2.8)			730 (11.9)		
<8 days out of 10 days	10,565	3,400 (3,120, 3,680)		569 (5.4)			1,242 (11.8)		
None	2,810	3,390 (3,110, 3,670)		322 (11.5)			350 (12.5)		

* *Using the Chi-square test for trend test*.

The results of multivariate generalized linear models on the relationship between periconceptional folic acid supplementation and newborn birth weight are shown in Table 5. The results of the generalized linear model showed that after controlling for confounding factors including maternal ethnicity, age, education level, occupation, household registration, parity, delivery year, pre-pregnancy BMI, gender of newborns, gestational diabetes, hypertensive disorders of pregnancy, thyroid disease, liver disease and anemia, no statistical correlation was found between the users and non-users of folic acid supplements, the initiation of supplements being pre or post conception, or high or low compliance of taking supplements with the birth weight. However, babies born to mothers who took MMFA had a higher median birth weight than babies whose mothers took FAO, according to both a univariate analysis *(P* < 0.01) and multivariate analysis β = 15.09 (*95% CI*: 4.92–24.73).

**Table 5:** Association of periconceptional of folic acid supplementation and birth weight of newborns as well as risk of SGA and LGA evaluated with multivariate models.

**Folic acid supplementation**	**Generalized linear model for birth weight**			**Logistic regression model for risk**	
	**β (95% CI)**	**Waldχ^2^**	***P*-value**	**SGA, a*OR* (95%, *CI*)**	**LGA, a*OR* (95%, *CI*)**
Folic acid supplementation					
Yes	9.64 (−6.51, 25.80)	1.37	0.242	0.81 (0.68, 0.97)	0.94(0.83, 1.06)
No	0			1	1
Formula					
MMFA	15.09 (4.92, 24.73)	9.40	0.002	0.96 (0.86, 1.07)	1.07 (0.99, 1.16)
FAO	0			1	1
Initiation time					
FAO
Before conception	−10.55 (−25.92, 4.82)	1.81	0.178	1.02 (0.86, 1.21)	1.06 (0.94, 1.21)
After conception	0			1	1
MMFA					
Before conception	3.94 (−8.95, 16.84)	0.36	0.549	0.92 (0.80, 1.07)	1.02 (0.92, 1.13)
After conception	0			1	1
Supplement frequency					
FAO					
8 days or more out of 10 days	−1.73 (−20.61, 17.16)	0.03	0.858	0.83(0.67, 1.01)	0.93 (0.80, 1.08)
<8 days out of 10 days	3.23 (−15.62, 22.08)	0.11	0.737	0.84 (0.69, 1.03)	0.88 (0.76, 1.02)
None	0			1	1
MMFA					
8 days or more out of 10 days	25.59 (6.49, 44.69)	6.90	0.009	0.74 (0.60, 0.91)	1.00 (0.86, 1.16)
<8 days out of 10 days	10.53 (−6.91, 27.97)	1.40	0.236	0.84 (0.70, 1.02)	0.94 (0.82, 1.08)
None	0			1	1

*Adjusted confounding factors: Ethnicity, Age, Education level, Occupation, Household registration, Parity, Delivery year, Pre-pregnancy BMI, Gender of newborns, Gestational diabetes, Hypertensive disorders of pregnancy, Thyroid disease, Anemia, Liver disease*.

Compared with newborns whose mothers did not use any folic acid supplements, newborns with maternal periconceptional folic acid supplementation had a lower risk of SGA [(*aOR*) = 0.81 (95% *CI*: 0.68–0.97)]. Babies born to mothers who took MMFA with high compliance had a 25.59 g (95% *CI:* 6.49–44.69) higher median birth weight than those whose mothers did not take any supplements. After adjusting for confounding factors, the risk of SGA among newborns whose mothers took FAO or MMFA during the periconceptional period with high compliance was lower than those whose mothers did not take any folic acid supplements, with *aOR* = 0.83 (95% *CI:* 0.67–1.01) and *aOR* = 0.74 (95% *CI*: 0.60–0.91) for FAO and MMFA, respectively.

### Comment

This study was based on the pregnancy registry data from a population-based prenatal health care system. We found that periconceptional FAO supplementation has no impact on the average birth weight of offspring and the risk of LGA, but compared with FAO, MMFA supplementation increases average birth weight, and highly compliant use of FAO or MMFA is associated with reduced the risk of SGA, with MMFA having a stronger effection than FAO.

Previous studies investigating the associations of folic acid supplementation with the birth weight of offspring provided inconsistent results (19–21). Variations in the formula of folic acid supplements, the initial time and duration of folic acid supplementation, and lack of consideration of the effects of preterm birth and parity on birth weight among different populations might be among the reasons for these inconsistent results.

Our study confirmed that women taking MMFA during the periconceptional period can increase the average birth weight of offspring by 25.59 g, while this association was not found for women taking FAO. The median birth weight of the offspring of women with highly compliant MMFA supplementation was higher than that of women without supplementation, but this association was not found in women with a low compliance of MMFA supplementation. We speculate that there may be several reasons for the observed association. First, there is a dose-response relationship between folic acid content and the birth weight of their offspring. The folic acid content of FAO is generally 0.4 mg in China, while the folic acid content of MMFA is often 0.8 mg, which explains why the supplementation of MMFA increases the average birth weight of offspring, while supplementation of FAO had no such effect. The total folic acid intake in the MMFA supplement group with high compliance was higher than that in the low compliance group, which explains why the average birth weight of the offspring supplemented with high compliance MMFA was higher than that of the women without folic acid supplement, while there was no such effect in the low compliance MMFA supplement group. Fekete's (26) study had similar results to ours: a 2% increase in birth weight for every 2-fold increase in folic acid intake. Secondly, other nutrients in MMFA may have an effect on the birth weight of their offspring, such as iron (27), calcium (28), selenium (29), and others. These speculations still require further demonstration.

We found that FAO or MMFA supplementation during the periconceptional period can significantly reduce the risk of SGA, but not LGA. Li et al. (30) reached a similar conclusion, finding that folic acid supplementation during the periconceptional period has a greater effect on lighter newborns, while heavier newborns experience little or no effect. We hypothesized that periconceptional folic acid supplementation can prevent the fetus from having a low birth weight resulting from maternal folic acid deficiency, while the birth weight may not be further improved when the added exogenous folic acid exceeds the optimal requirement.

Unexpectedly, our study showed that the protective association of folic acid supplements against SGA birth occurred whether the mother initiated taking the supplements pre or post conception. Our conclusion is not completely consistent with the conclusion of the 1999–2012 Jiaxing Birth Cohort study (31), which found that folic acid supplementation before conception was associated with a reduced risk of SGA births, while folic acid supplementation after conception was not. Compared with the Jiaxing Birth Cohort study, this study had fewer confounding factors such as diet and lifestyle changes caused by China's economic and social development. In addition, due to the promotion of the government's free folic acid supplement policy, the prevalence of periconceptional folic acid supplementation among the women in our study was much higher than that of the Jiaxing Birth Cohort study (90.9 vs. 24.9%), which may also be the reason for the inconsistent results.

In addition, our study also found that periconceptional use of FAO or MMFA had a protective effect against the risk of SGA at high compliance, but not at low compliance. The possible explanation for this association is that, on the one hand, there may be the same dose-response effect; on the other hand, women who take folic acid supplements with high compliance, may also be more health-conscious and more associated with healthier lifestyles or better economic conditions, thus strengthening the inverse association between folic acid supplements and the risk of SGA.

The mechanisms behind the relationship between folic acid supplementation and the birth weights of offspring are not fully understood. The first trimester of pregnancy is a critical period for the rapid development of fetal organ systems, thus the status of maternal nutrition during the periconceptional period plays an important role in fetal growth and development (32, 33), especially because it may affect the epigenetic mechanism of the placenta and fetus (34, 35). Therefore, folic acid supplementation during the periconceptional period can benefit women with low folate levels. However, while folic acid is necessary for one-carbon metabolism and is essential to the biosynthesis and methylation of DNA and RNA, relatively little is known about the implications of maternal folate status during the periconceptional period of pregnancy for fetal growth. We made two hypotheses. First, the mother can transfer folate to the fetus against the concentration gradient (36). If maternal low folate status leads to an increase in plasma homocysteine levels, this may increase the risk of placental vascular endothelial injury and exchange dysfunction, thereby disturbing the transport of nutrients and oxygen to the fetus (37, 38). Second, folic acid is an important methylated donor. Some studies have shown that changes in folic acid exposure during periconception may lead to epigenetic changes in the genomes of offspring, which in turn affects fetal development and birth weight (34, 35). However, these hypotheses still require detailed further study.

In general, our study has several strengths. First, our study included a variety of variables for the manner of taking folic acid supplements, including the formula, compliance, and the initial timing of folic acid supplementation, which resulted in a more comprehensive classification of the exposure and better revealed the association between folic acid supplementation and birth weight or the risk of SGA and LGA in the Chinese population. Second, this study had a large sample size. The folic acid supplementation information data was collected during the 1st trimester of pregnancy, which decreases the influence of recall bias compared with information collected after delivery. Third, our research data had strict quality control (as described in the methods section). Finally, 94% of the participants were urban women of Han ethnicity having similar dietary patterns and lifestyles, which further reduced the remaining confounding factors.

Despite the strengths of our study, we recognize that our study had several limitations. First, we did not collect information on the dosages of folic acid in the supplement pills, which is a major limitation of the present study. Whereas, the dosage of the folic acid pill on the Chinese market is 0.4 mg per tablet, for MMFA the folic acid dosages are different, although 80% of the pills sold are one main brand with a dosage of 0.8 mg of folic acid per tablet. The data was collected when the women in their 1st trimester of gestational period in the community hospital through face to face interview by the doctors and nurses. Based on the data we have conducted other research previously (26) Second, the PHCS in Beijing did not provide enough detailed information on potential confounding factors, including lifestyle factors and physical activity, thus they could not be controlled for in this study. Third, the sample size was disparity between FA users (28.297 women) and non-users (2.810 women) in this study. To evaluate the impact on the results we calculated the sample size and concluded that the results of the study are credible. According to the WHO Multi-Country Survey on Maternal and Newborn Health Research Network, the incidence of SGA in China was 10.2% (39). Our previous showed that the rate of maternal periconceptional folic acid supplementation was 90.08% in Tongzhou of Beijing (18), that is, the sample size ratio for the none-used group to the folic acid used group was 1:9. And the SGA rates ration (*RR*) for folic acid supplementation vs none supplementation was 0.72 in a birth cohort study in Lanzhou city in north of China (40). The sample size is 1,100 for none folic acid used group and 10,991 for folic acid supplemented group, with α = 0.05 (two-sided test), β = 0.1, calculated with PASS (Ver.11.0) model “Two Independent Proportions (Null Case) Power Analysis”. So the power should be over 0.9 under the sample size in this study. Fourth, we did not account for genetic factors, such as MTHFR gene polymorphism, which can cause differences in the ability to utilize folic acid, even under the same dosage (41, 42). Finally, this study only used the data of folic acid taken by women during the periconceptional period, and lacks information on supplementation during the second and third trimester of pregnancy, thus it cannot fully reflect the impact of maternal folic acid supplementation on the weight of newborns.

## Conclusion

Periconceptional FAO supplementation has no impact on the median birth weight of offspring and the risk of LGA. Compared with FAO, MMFA supplementation increases the average birth weight, and a high compliance of supplementation with FAO or MMFA may reduce the risk of SGA, with MMFA having ad stronger effect than FAO.

This study shows that periconceptional FAO supplementation has no impact on the average birth weight of offspring and the risk of LGA, but compared with FAO, MMFA supplementation may increase average birth weight, and highly compliant supplementation with MMFA may reduce the risk of SGA. Our findings highlight the positive effect of folic acid supplementation on birth weight and emphasize the necessity and safety of folic acid supplementation for women during the periconceptional period.

## Data Availability Statement

The original contributions presented in the study are included in the article/supplementary material, further inquiries can be directed to the corresponding author/s.

## Ethics Statement

This study was approved by the Institutional Review Board of Peking University (No.: IRB00001052-18010). The research proposal was approved by the Institutional Review Board of Peking University, because as it was a retrospective study based on routine data, we need not to get informed consent from the participants. This manuscript reported adherence to Declaration of Helsinki. The patients/participants provided their written informed consent to participate in this study.

## Author Contributions

JL and CW: data curation, methodology, and writing—original draft. JZ, SL, and MT: data curation. LJ and WM: collected data and contributed to data cleaning and analysis. AR: critically reviewed the manuscript for important intellectual content. LJ and LC: conceptualization, funding acquisition, project administration, writing—review, and editing. All authors contributed to the article and approved the submitted version.

## Funding

This work was supported by Capital's Funds for Health Improvement and Research, CFH) in People's Republic of China (020-1-5112). The funder of the study had no role in study design, data collection, data analysis, data interpretation, or writing of the report. The corresponding 10 authors had full access to all the data in the study and had final responsibility 10 for the decision to submit for publication.

## Conflict of Interest

The authors declare that the research was conducted in the absence of any commercial or financial relationships that could be construed as a potential conflict of interest.

## Publisher's Note

All claims expressed in this article are solely those of the authors and do not necessarily represent those of their affiliated organizations, or those of the publisher, the editors and the reviewers. Any product that may be evaluated in this article, or claim that may be made by its manufacturer, is not guaranteed or endorsed by the publisher.

## Abbreviations

FAO: folic acid only
MMFA: multiple micronutrients with folic acid
SGA: small for gestational week age
LGA: large for gestational week age
LMP: last menstrual period
BMI: body mass index
PHCS: Prenatal Health Care System.
